# Dose-Response Relationship between Dietary Magnesium Intake and Risk of Type 2 Diabetes Mellitus: A Systematic Review and Meta-Regression Analysis of Prospective Cohort Studies

**DOI:** 10.3390/nu8110739

**Published:** 2016-11-19

**Authors:** Xin Fang, Hedong Han, Mei Li, Chun Liang, Zhongjie Fan, Jan Aaseth, Jia He, Scott Montgomery, Yang Cao

**Affiliations:** 1 Unit of Biostatistics, Institute of Environmental Medicine, Karolinska Institutet, Stockholm 17177, Sweden; yang.cao@ki.se; 2 Department of Health Statistics, Second Military Medical University, Shanghai 200433, China; he_dong1102@126.com; 3 Department of Cardiology, Shanghai Changzheng Hospital, Second Military Medical University, Shanghai 200003, China; happyxiaoant@126.com (M.L.); chunliangliang@hotmail.com (C.L.); 4 Department of Cardiology, Peking Union Medical College Hospital, Peking Union Medical College, Chinese Academy of Medical Sciences, Beijing 100730, China; Fan@pumch.cn; 5 Faculty of Public Health, Hedmark University of Applied Sciences, Elverum 2411, Norway; jaol-aas@online.no; 6 Innlandet Hospital Trust, Kongsvinger Hospital Division, Kongsvinger 2226, Norway; 7 Clinical Epidemiology and Biostatistics, School of Medical Sciences, Örebro University, Örebro 70182, Sweden; scott.montgomery@regionorebrolan.se; 8 Clinical Epidemiology Unit, Karolinska University Hospital, Karolinska Institutet, Stockholm 17177, Sweden; 9 Department of Epidemiology and Public Health, University College London, London WC1E 6BT, UK

**Keywords:** magnesium, dietary intake, type 2 diabetes, prospective study, cohort study, meta-analysis

## Abstract

The epidemiological evidence for a dose-response relationship between magnesium intake and risk of type 2 diabetes mellitus (T2D) is sparse. The aim of the study was to summarize the evidence for the association of dietary magnesium intake with risk of T2D and evaluate the dose-response relationship. We conducted a systematic review and meta-analysis of prospective cohort studies that reported dietary magnesium intake and risk of incident T2D. We identified relevant studies by searching major scientific literature databases and grey literature resources from their inception to February 2016. We included cohort studies that provided risk ratios, i.e., relative risks (RRs), odds ratios (ORs) or hazard ratios (HRs), for T2D. Linear dose-response relationships were assessed using random-effects meta-regression. Potential nonlinear associations were evaluated using restricted cubic splines. A total of 25 studies met the eligibility criteria. These studies comprised 637,922 individuals including 26,828 with a T2D diagnosis. Compared with the lowest magnesium consumption group in the population, the risk of T2D was reduced by 17% across all the studies; 19% in women and 16% in men. A statistically significant linear dose-response relationship was found between incremental magnesium intake and T2D risk. After adjusting for age and body mass index, the risk of T2D incidence was reduced by 8%–13% for per 100 mg/day increment in dietary magnesium intake. There was no evidence to support a nonlinear dose-response relationship between dietary magnesium intake and T2D risk. The combined data supports a role for magnesium in reducing risk of T2D, with a statistically significant linear dose-response pattern within the reference dose range of dietary intake among Asian and US populations. The evidence from Europe and black people is limited and more prospective studies are needed for the two subgroups.

## 1. Introduction

Type 2 diabetes mellitus (T2D) represents a growing public health burden across the world and is a leading cause of death. In 2013, an estimated 340 million people worldwide had T2D and this number is expected to increase to 400 million or more by 2030 [1,2]. Obesity and diet are widely believed to play an important role in the development of T2D [3,4]. Magnesium is the most abundant divalent intracellular cation, the second most abundant cellular ion next to potassium and the fourth cation in general in the human body. Of the 21–28 g of magnesium present in the adult human body, 99% is distributed in the intracellular compartment, and only 1% in the extracellular fluid [5]. Magnesium has received considerable interest for its potential in improving insulin sensitivity and preventing diabetes [6,7,8,9]. T2D is often accompanied by altered magnesium status. An increased prevalence of magnesium deficit has been identified in T2D patients, especially in those with poorly controlled glycemic profiles, longer duration of disease and the presence of micro- and macro-vascular chronic complications [10,11,12]. A number of prospective cohort studies of magnesium intake and diabetes incidence have been conducted [7,13,14,15,16,17,18,19,20,21,22,23,24] and statistically significant negative associations between magnesium intake and risk of T2D were reported in previous meta-analyses [25,26,27]. However, these meta-analyses did not examine whether the association was confounded by other established risk factors such as being overweight and other factors highly associated with magnesium intake, such as amount of cereal fiber, and whether the relationship is linear.

During the past few years, the number of studies on this topic has increased. With mounting evidence, we conducted a meta-analysis of prospective cohort studies for the following purpose: (1) to update the epidemiological evidence on the association between magnesium intake and T2D risk; (2) to evaluate the association according to characteristics of study designs and population; and (3) to examine the linear and nonlinear dose-response pattern of magnesium intake and T2D risk.

## 2. Materials and Methods

The protocol for this systematic review was registered in the PROSPERO database of prospectively registered systematic reviews in February 2016 (www.crd.york.ac.uk/PROSPERO; CRD42016033519). The completed review conforms to the standard criteria PRISMA (Preferred Reporting Items for Systematic Reviews and Meta-Analysis) and MOOSE (Meta-analysis of Observational Studies in Epidemiology) [28,29].

### 2.1. Data Sources and Searches

We conducted a systematic review for all population-based studies that evaluated the association of magnesium intake with T2D. We searched Pubmed (http://www.ncbi.nlm.nih.gov/), Web of Science (http://webofscience.com/), ScienceDirect (http://www.sciencedirect.com/) and China Knowledge Resource Integrated Database (http://oversea.cnki.net/kns55/default.aspx) and the Cochrane Library (http://www.cochranelibrary.com/) from their inception to 29 February, 2016. The later cut-off date to 30 June 2016 was subsequently revised to include the latest published studies. To avoid publication bias, we also used the National Library of Medicine Gateway (https://gateway.nlm.nih.gov/), Virtual Health Library (http://pesquisa.bvsalud.org/portal/), the System for Information on Grey Literature in Europe (http://www.opengrey.eu/), the National Academic Research and Collaborations Information System (http://www.narcis.nl/?Language = en) and Grey Literature Report (www.greylit.org) to find potential unpublished relevant studies. Key search terms included magnesium intake, type 2 diabetes, diabetes mellitus, prospective study, longitudinal study, cohort study, and nested case-control study, combined with incidence or risk. These searches were supplemented by hand-searching of the reference lists of identified research articles or relevant reviews. No language restrictions were imposed.

### 2.2. Inclusion Criteria

We only included original research in this meta-analysis. Reviews, editorials, commentaries and letters were not eligible. All population-based cohort studies (including nested case-control studies) were included if they fulfilled the following criteria: (1) had a prospective study design; (2) the doses of magnesium intake (dietary and supplemental) were reported; (3) the endpoint of interest was incidence of T2D; (4) the risk ratio was reported such as relative risk (RR), odds ratio (OR) or hazard ratio (HR), as well as the associated 95% confidence interval (CI) or other data to estimate the variance or accuracy (standard deviation or standard error) were reported; (5) the risk assessment had to be adjusted for potential confounding factors or by other forms of standardization (if applicable). For multiple studies using the same population, only the study with the largest number of events or with adjustment for additional potential confounders was included. Studies were excluded if they: (1) focused on the populations with disrupted mineral homeostasis (such as patients with heart failure or kidney disease); (2) were narrative reviews, editorial papers, methodological papers, experimental studies, case control or cross-sectional; (3) assessed type 1 diabetes; (4) identified a dietary pattern that did not fit into healthy or unhealthy dietary pattern categories; (5) evaluated magnesium only in drinking water or had no reliable magnesium estimates. For included studies only in abstract form, we tried to contact authors to obtain the necessary estimates or risks and relevant accuracy.

### 2.3. Quality Assessment and Data Extraction

Computerized bibliographic searches of pre-determined literature databases used an optimized version of the Cochrane Collaboration search strategy [30]. Three investigators (X.F., C.L. and M.L.) screened all the identified titles and abstracts for relevance (*n* = 2858). Full papers were downloaded for all the abstracts judged potentially relevant (*n* = 60). No new studies were identified among the cited references of all included articles. Of 60 full-text articles reviewed independently, we excluded 35 studies for the following reasons: they were not prospective studies (*n* = 14); outcomes were not T2D (*n* = 9); did not report dietary magnesium (*n* = 7); did not assess the risk (*n* = 3); or duplicated another study (*n* = 1). All papers identified through the screening process were assessed for relevance independently by two investigators (C.L. and M.L.) using standardized study assessment and a sorting form. The studies were evaluated and scored based on the guidelines adapted from the tools for assessing quality and susceptibility to bias in observational studies in epidemiology [31]. Inter-rater agreement was substantial (Cohen κ > 0.6) [32]. No studies were excluded by the quality assessment. In total, 25 studies met the inclusion criteria and were included in the meta-analysis.

Full papers were obtained for all abstracts judged potentially relevant. Data extraction was conducted independently by two investigators (X.F. and M.L.) with the use of a standardized electronic form in Microsoft Excel. The following data were extracted from each study: first author’s surname; study design; location; year the study started, finished and was published; age; sex; ethnicity; sample size (number of those with T2D and the total number of participants); diseases present at baseline (hypertension or hypercholesterolemia, etc.); magnesium intake modes (dietary or supplemental) and dose; as well as covariates adjusted for in the multivariable analysis. For magnesium intake, data on assessment method used (food frequency questionnaire, dietary recall, other) and whether the data were energy-adjusted (yes, no) were obtained. For each study, the median magnesium intake for each quantile (tertile, quartile or quintile) of magnesium intake was assigned as the representative dose. When the median intake per quantile was not provided, we assigned the midpoint of lower and upper boundaries in each quantile as the average intake. If the lower or upper boundary for the lowest or highest quantile, respectively, was not reported, we assumed that the boundary has the same amplitude as the closest quantile. The increment of dietary magnesium intake was calculated as the difference between the representative dose of the higher quantiles and the representative dose of the control quantile.

For each dose quantile, we extracted RR, OR or HR with their measure of uncertainty (standard error) or variance (95% CI). Risk estimates for continuous exposure were also extracted. If estimates were presented for more than one multivariate model, we only extracted estimates from the model maximally adjusted for potential confounding variables to ensure a conservative conclusion. Because there are studies based on the same cohort but conducted at different times, they shared the T2D patients. When we calculated the total participants and T2D cases, we only used the studies with the largest numbers.

### 2.4. Statistical Analysis

We used OR and HR as RR in our pooled analysis because when event rates are small, the OR, HR and RR approximate one another [33]. We estimated a pooled risk with 95% CI for a 100 mg/day increase in daily magnesium intake for the studies. To maximize all the data for calculating the pooled dose-response, the restricted maximum likelihood (REML) approach proposed by Harbord [34], which provides improved estimation of the between-study variance, was used to compute the linear trend of the log transformed risk estimates across magnesium intake doses. We also performed subgroup analysis by level of magnesium intake increment, sex, geographic area and adjustment.

The Higgins’s *I*^2^ statistic, a quantitative measure of inconsistency, was calculated to evaluate the statistical heterogeneity across the studies [35]. *I*^2^ > 30% was considered as at least moderate heterogeneity. In view of substantial heterogeneity being detected, we presented the pooled estimates based on the random-effects model.

Potential publication bias was assessed by Egger’s test [36]. Because the sample sizes of reference groups and comparative groups were balanced in all the studies, we used Harbord’s modification to Egger’s test to reduce the false-positive rate [37]. The results were also confirmed by Begg’s test [38] and Peters’s test [39].

Potential nonlinear associations were assessed using restricted cubic splines; we used four knots at fixed percentiles 5%, 35%, 65% and 95% of the distribution [40]. The study-specific estimates were pooled by using the REML method in a random-effects meta-analysis [41].

We also conducted a sensitivity analysis to investigate the influence of a single study on the overall risk estimate by dropping one study in each turn. We performed all analyses in Stata (version 14.1; Stata Corp., College Station, TX, USA). A *p* value < 0.05 was considered statistically significant, except where otherwise specified.

## 3. Results

### 3.1. Eligible Studies and Characteristics

**Figure 1:**
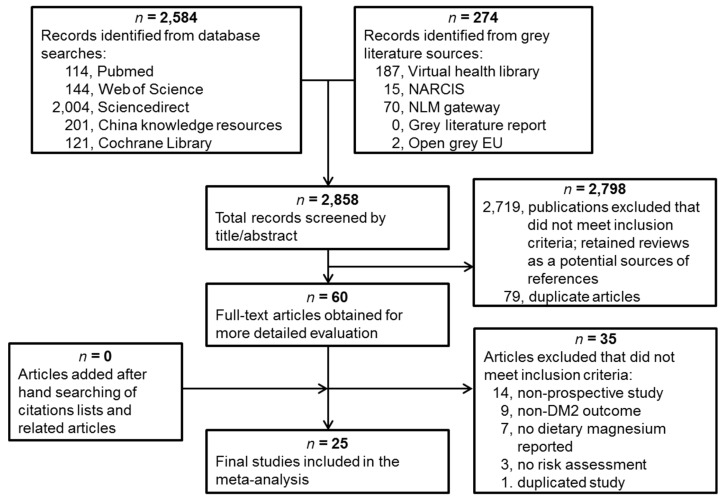
Screening and selection of articles on dietary magnesium intake and risk of type 2 diabetes mellitus.

Our literature search identified 25 studies from 17 cohorts that met the eligibility criteria (Figure 1). These studies were published between 1997 and 2014 and comprised 637,922 individuals and 26,828 T2D cases after excluding duplicated cohorts (Table 1). There were 16 studies conducted in the U.S. (including Hawaii), two in Europe (Italy and Germany), and seven in Asia (five in Japan and two in China). Studies treated dietary calcium [42,43,44], red/processed meat [45,46], whole grain [47], fiber [14,15,48], vitamin D [43,44], carbohydrates [14], coffee [49] or glycemic load [15,48,50] as main exposures, but also reported dietary magnesium intakes which were included in our meta-analysis. The main endpoints of two studies were impaired insulin metabolism [7] and insulin resistance [22], but both studies also reported the incidence of T2D. Participants were predominately middle-aged at baseline, with a mean age of 51.2 years and a mean BMI of 25.0 kg/m^2^ across the studies. The length of the follow-up period ranged from four to 20 years.

Dietary intake of magnesium was evaluated by food frequency questionnaires (FFQs) in all the studies and 13 studies indicated that the questionnaires were validated. The median magnesium intake of the different dose groups ranged from 115 mg/day in U.S. black women [18] (much lower than the US Recommended Dietary Allowance of 400 mg/day for men and 310 magnesium for women >30 years [51]) to 478 mg/day in a U.S. population [22]. T2D was ascertained by self-report and 21 studies indicated that the self-reported diagnoses were validated.

For the 16 studies with the magnesium as the main exposure, although the degree of covariate and confounder adjustment varied in the multivariate models, most studies adjusted for age, body mass index (BMI), total energy intake, smoking, physical activity, family history of diabetes and hypertension; fewer studies adjusted for intake of calcium or other nutrition supplement and education attainment. For the nine studies with other nutrients as main exposure, only crude RRs were extracted.

### 3.2. Dietary Magnesium Intake and Type 2 Diabetes Mellitus (T2D) Incidence

**Figure 2:**
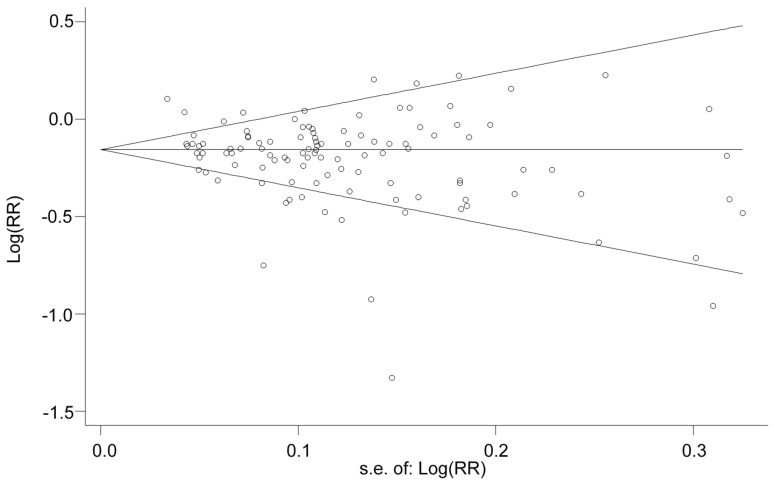
Funnel plot with pseudo 95% confidence limits.

We divided the increment of dietary magnesium intake into four categories, i.e., <50 mg/day, 50–99 mg/day, 100–149 mg/day and ≥150 mg/day, by subtracting the reference doses from the compared doses. Heterogeneity was found by Higgins’s test, with *I*^2^ = 73.3% (*p* < 0.001) for all compared doses, and 67.2% (*p* < 0.001), 75.0% (*p* < 0.001), 52.3% (*p* = 0.005) and 54.5% (*p* = 0.031) for four increment categories, respectively. However, the approximately symmetric funnel plot of all but four doses suggests a moderate homogeneity among the studies (Figure 2). Although there is evidence of publication bias among all compared doses for Egger’s test (*p* = 0.002), Begg’s, Harbord’s and Peters’s tests show no evidence of publication bias (*p* = 0.170, 0.401 and 0.105, respectively).

The overall combined RR for T2D incidence is 0.83 (95% CI: 0.80, 0.86; *p* < 0.001) for all compared doses. The results of subgroup analysis are presented in Table 2. A statistically significant negative association between dietary magnesium and risk of T2D incidence was observed across sexes and the pooled RRs are 0.81 (95% CI: 0.77, 0.86) for women, 0.84 (95% CI: 0.80, 0.88) for men, and 0.85 (95% CI: 0.78, 0.94) for the studies that only reported sex-combined risk estimates. The association was statistically significant in all the study areas and the largest magnitude association was found among U.S. studies (pooled RR = 0.82 in U.S. vs. 0.86 in Europe and 0.85 in Asia), compared with the unadjusted associations (pooled RR = 0.81; 95% CI: 0.74, 0.88), with lower magnitude after adjustment (pooled RR = 0.83; 95% CI: 0.81, 0.86). Two studies investigated the association specially in black people and showed a statistically significant association (pooled RR = 0.82; 95% CI: 0.71, 0.94), however, it seems this is mainly observed among black women [18] rather than black men [13].

**Figure 3:**
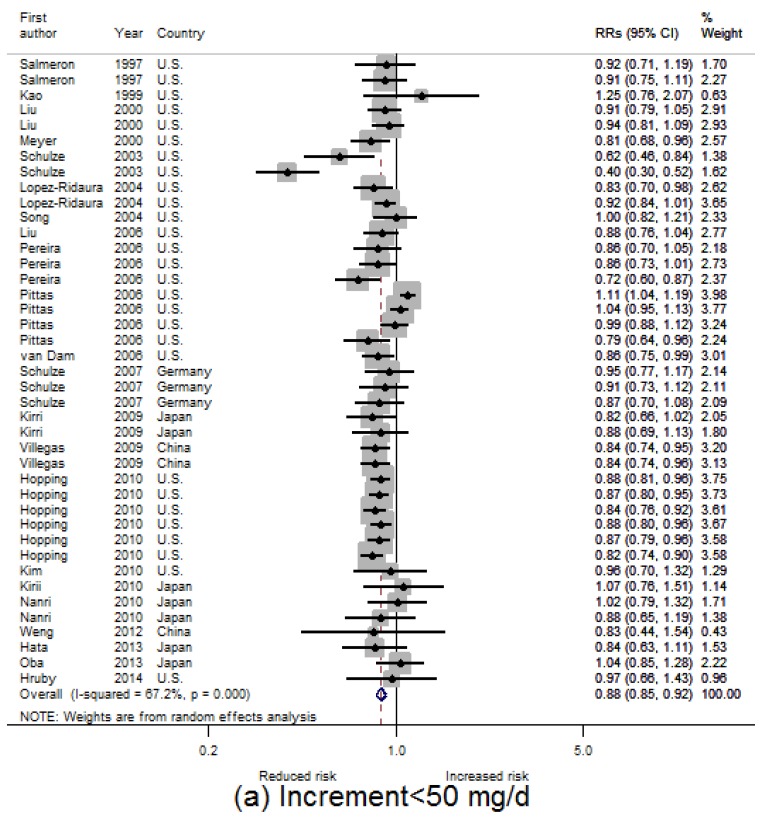

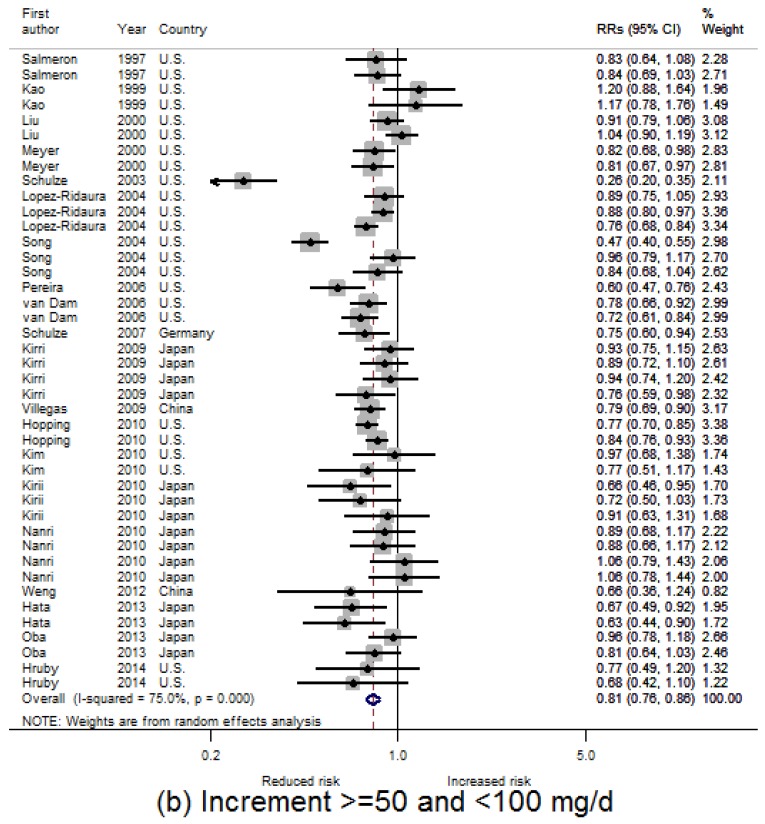

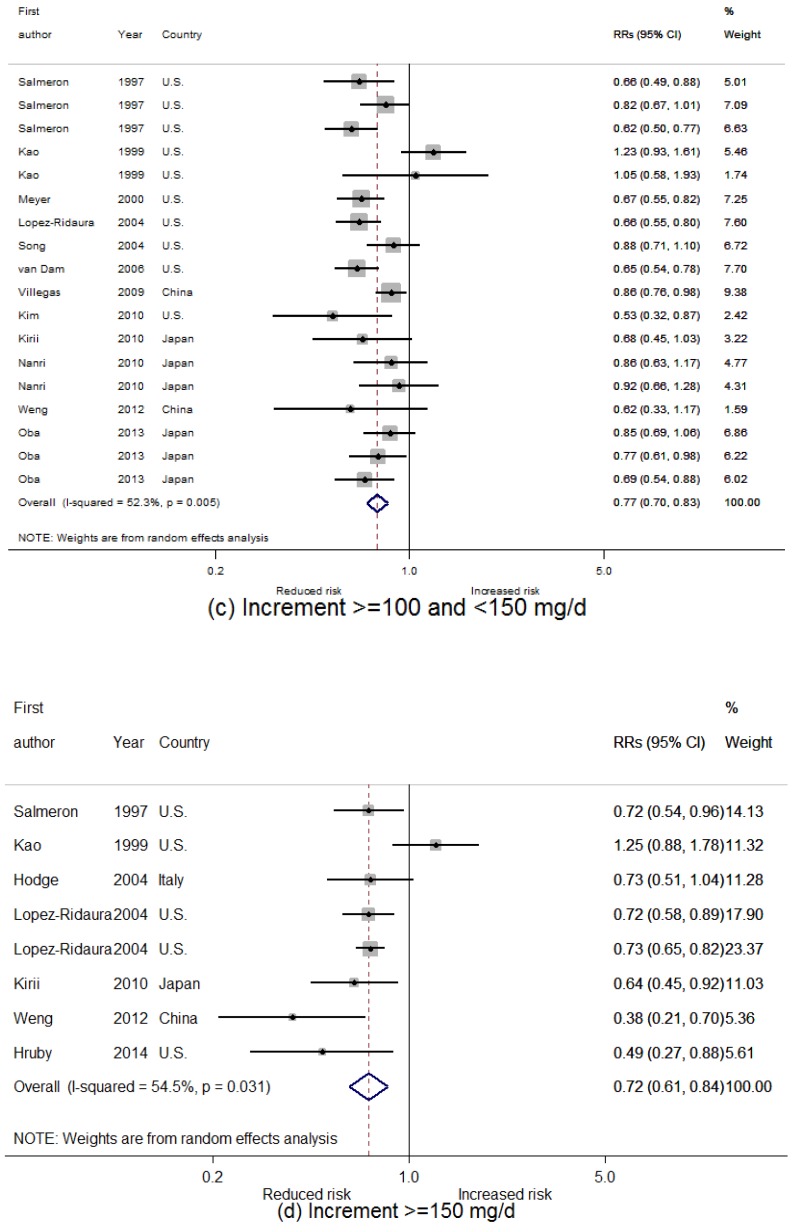
Relative risks (RRs) for risk of T2D incidence for different dietary magnesium increment categories: (**a**) <50 mg/day; (**b**) 50–99 mg/day; (**c**) 100–159 mg/day; (**d**) ≥150 mg/day.

The dose-category-specific pooled RRs for T2D incidence from the included studies are shown in Figure 3a–d, which are 0.88 (95% CI: 0.85, 0.92), 0.81 (95% CI: 0.76, 0.86), 0.77 (95% CI: 0.70, 0.83) and 0.72 (95% CI: 0.61, 0.84) for increment <50 mg/day, 50–99 mg/day, 100–149 mg/day and ≥150 mg/day, respectively. In general, the RR decreases 4% to 7% per 50 mg/day increment (equivalent to 8% to 14% per 100 mg/day increment) in dietary magnesium intake.

### 3.3. Linear Dose-Response Relationship

**Figure 4:**
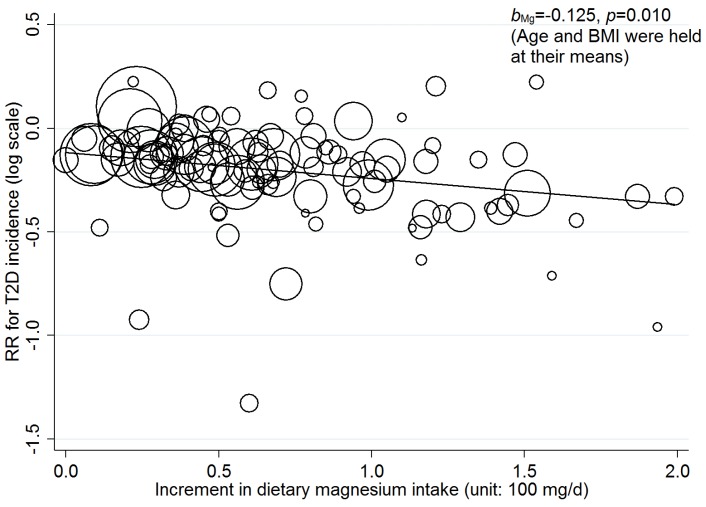
Dose-response relationship between risk of T2D incidence and incremental dietary magnesium intake (excluding one extreme dose). The size of the bubble reflects the study-specific analytical weight, i.e., the inverse of the variance.

After adjusting for age and BMI in random-effects meta-regression models, a statistically significant linear dose-response relationship between incremental dietary magnesium intake and T2D incidence was found across all the studies (see Table 3 and Figure 4). The RRs (95% CI) for the association of a 100 mg/day increment in dietary magnesium intake with T2D incidence are 0.92 (95% CI: 0.85, 0.99) and 0.88 (0.80, 0.97) for including and excluding one extreme dose, respectively. The statistically significant linear dose-response relationship was also found for men (RR = 0.87; 95% CI: 0.77, 0.98) but not for women (RR = 0.88; 95% CI = 0.76, 1.02). Regarding study areas, significantly linear dose-response relationship was only found in Asia (RR = 0.87; 95% CI: 0.77, 0.98). No significant linear dose-response relationship was found in black people (RR = 0.75; 95% CI = 0.23, 2.41).

In general, the risk of T2D incidence decreases by 8% (across all studies) to 13% (in the Asian population) per 100 mg/day increment in dietary magnesium intake, which is consistent with the result from dose-category-specific analysis.

### 3.4. Nonlinear Dose-Response Relationship

**Figure 5:**
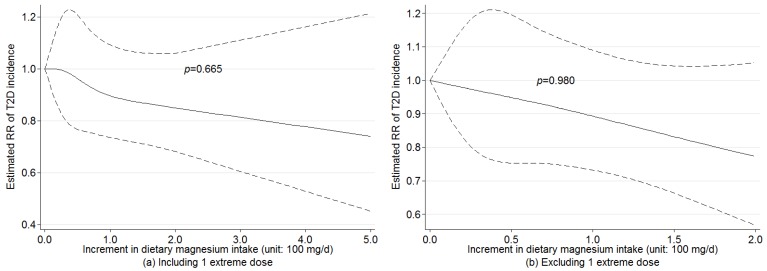
Examination of nonlinear association between increment in dietary magnesium intake and risk of T2D incidence by random-effects model with the use of restricted cubic splines.

We found no evidence of nonlinear associations between dietary magnesium intake and T2D incidence across all the studies with (*p* = 0.665) or without (*p* = 0.980) one extreme dose (Figure 5), adjusting for age and BMI. For subgroup analysis, no evidence of nonlinear association was found for women (*p* = 0.637), men (*p* = 0.790), sex-combined (*p* = 0.987), black people (*p* = 0.787), U.S. population (*p* = 0.686), Asian population (*p* = 0.519), adjusted RRs (*p* = 0.663) and crude RRs (*p* = 0.250), which suggested that pooling the dose-response estimates from linear trend estimation for dietary magnesium intake and T2D incidence was appropriate. Because of insufficient dose observations, no nonlinear association was evaluated for European studies.

### 3.5. Sensitivity Analysis

Regarding the combined risk of T2D incidence for all studies, the sensitivity analysis omitting one study at a time yielded statistically significant RRs within a very narrow range from 0.82 (95% CI: 0.79, 0.84) to 0.84 (95% CI: 0.81, 0.87). The subgroup analyses also showed robust results for women (RR range: 0.80, 0.84), men (RR range: 0.83, 0.85), sex-combined (RR range: 0.79, 0.88), U.S. population (RR range: 0.80, 0.84), Asian population (RR range: 0.83, 0.85), adjusted RRs (RR range: 0.82, 0.84) and crude RRs (RR range: 0.78, 0.86). However, because of a limited number of studies in Europe and black people, the sensitivity analysis generated relatively wide ranges for these two subgroups. The RR ranges were from 0.73 (95% CI: 0.51, 1.04) to 0.87 (95% CI: 0.78, 0.97) and from 0.76 (95% CI: 0.67, 0.85) to 1.17 (95% CI: 0.88, 1.54) for European studies and black people, respectively.

The sensitivity analyses for linear and nonlinear dose-response relationships between incremental dietary magnesium intake and the risk of T2D incidence show similar results. The RRs of linear dose-response relationship across all the studies are statistically significant and range from 0.88 (95% CI: 0.80, 0.97) to 0.93 (95% CI: 0.87, 1.00) when omitting one study at a time. The *p*-values for a nonlinear dose-response relationship across all studies range from 0.52 to 0.96 when omitting one study at a time. The results for subgroup analysis also change little except for European studies and black people (data not show). Overall, the results from sensitivity analyses indicate the robustness of our findings.

## 4. Discussion

This meta-analysis of 25 prospective studies showed a statistically significant negative association between dietary magnesium intake and T2D incidence. Compared with the lowest dietary magnesium consumption groups in the populations, the risk of T2D could be reduced by 19% in women and 16% in men (Table 2). The largest reduction of risk was observed for the U.S. population (18%). A statistically significant linear dose-response relationship was found between incremental dietary magnesium intake and T2D incidence across all the studies, in male and Asian populations, adjusting for age and BMI. The risk of T2D was associated with a reduction of 8%–13% per 100 g/day increment in dietary magnesium intake. After adjusting for age and BMI, we did not find a statistically significant nonlinear dose-response relationship of incremental dietary magnesium intake with T2D risk. The present systematic review, which includes a total of 637,922 participants and 26,828 T2D cases, provides the most robust evidence to date of the linear dose-response relationship between incremental dietary magnesium intake across its physiological range and risk of T2D.

### 4.1. Dose-Response Association of Dietary Magnesium Intake with T2D Incidence

A putative protective effect of magnesium intake against T2D incidence has been reported previously [13,14]. The negative association between magnesium intake and T2D incidence is biologically plausible and may be partially explained by its influence on glucose metabolism, insulin sensitivity and insulin action [5,6]. However, there is no conclusive evidence for the beneficial dose of dietary magnesium. For example, a meta-analysis indicated that 300 mg/day of magnesium intake was the essential dose for preventing T2D [27]. A cross-sectional study concluded that more than 300 mg/day of magnesium intake might not improve insulin sensitivity and have no influence [55]. Evidence from a prospective study showed that increased intake of magnesium might provide more benefit to participants with magnesium deficiency, as magnesium deficiency and hypomagnesaemia have been associated with the development of insulin resistance [18]. Meta-analyses of magnesium supplementation have also revealed conflicting results. A review including 12 randomized controlled trials (RCTs) assessing the efficacy of magnesium supplementation on insulin sensitivity and glucose levels included studies yielding inconsistent results [56]. However, concerning the effect of dietary magnesium intake on T2D incidence, the previous meta-analyses appeared to reach a consensus [19,25,27]. A meta-analysis of eight cohort studies showed a significant negative association (RR = 0.77; 95% CI: 0.72, 0.84). Another meta-analysis of 13 prospective cohort studies detected a significant negative association between magnesium intake and risk of T2D (RR = 0.78; 95% CI: 0.73, 0.84). A more recent meta-analysis with a total of 539,735 participants and 25,252 incident diabetes cases also indicated that magnesium intake was associated with a significant lower risk of T2D (RR = 0.77; 95% CI: 0.71, 0.82). However, there is less conclusive evidence of dose-response relationship between dietary magnesium intake and risk of T2D incidence [26]. By combining results of seven cohort studies, Larsson et al. observed a statistically significant lower risk of T2D for 100 mg/day increase in magnesium intake (RR = 0.85; 95% CI: 0.79, 0.92). Dong et al. also found a linear dose-response relationship for every 100 mg/day increment in magnesium intake (RR = 0.86; 95% CI: 0.82, 0.89) [25]. In contrast, a nonlinear relationship (*p* = 0.003) between magnesium intake and type 2 diabetes was reported by Xu et al. [27].

Our meta-analysis with a larger number of people with T2D did support existence of a statistically significant linear dose-response relationship between increased dose of dietary magnesium intake and T2D incidence among all the participants, especially in males and in Asian populations. In addition, we found no evidence of nonlinear associations between dietary magnesium intake and T2D incidence across all the studies (*p* = 0.665). The magnitude of the effect for 100 mg/day increment in magnesium intake in this meta-analysis (8%–13% reduction in risk of T2D) is comparable to those from other meta-analyses (8%–21% reduction in risk of T2D).

The discrepant findings between women and men might be due to the influence of other factors than magnesium intake. For example, the influence of magnesium on T2D incidence in women may be potentially attenuated by changed endogenous sex hormones in postmenopausal women [57,58]; which could accelerate T2D development and counteract the potentially protective influence of magnesium. The discrepancy between the Asian population and non-Asian population needs further research. However, because of the limited number of the included studies in Europe, the discrepancy may have been accidental and a chance finding cannot be ruled out.

It should be noted that our sensitivity analysis revealed robust associations for linear and nonlinear dose-response analyses among all the participants.

### 4.2. Implication for Practice 

Magnesium is mainly consumed through diet, and low magnesium consumption is common worldwide. It has been estimated that magnesium intake in a normal Western diet is often inadequate for the body’s needs; in the United States, 67% of women and 64% of men consume inadequate amounts of magnesium [59]. For people aged more than 30, the recommended dietary allowance (RDA) of magnesium for men and women is 350 mg/day and 420 mg/day, respectively [51]. On the basis of the studies we have reviewed, current evidence from population-based prospective cohort studies support the recommendation for increasing dietary magnesium intake.

### 4.3. Strengths and Limitations

The population-based evidence on whether increased magnesium intake may reduce T2D incidence is still sparse. To our knowledge, this study is the largest meta-analysis that investigated the dose-response relationship between dietary magnesium intake and T2D risk. It has several strengths. First, our data providing a systematic review of prospective studies represent the best available evidence of how dietary magnesium intake may influence risk of T2D incidence. Because of the lagged and cumulative effects of exposure on outcome of chronic diseases, the dose-response relationship without reversed causality would be revealed only by prospective studies rather than cross-sectional or retrospective studies. In addition, the prospective studies also minimized recall and selection bias. Second, by combining all available doses in included studies across a wide range of exposure, we increased the validity of the dose-response estimates. Our studies included 141 dietary magnesium doses and 108 risk estimates, which enabled us to estimate both linear and nonlinear dose-response relationship with a high statistical power. Third, age and BMI were adjusted for in our meta-regression model and stratified analyses were used for sex, study areas and adjustment, which reduced the potential confounding from demographic and other factors. Furthermore, the random-effects model considered the heterogeneity among studies, which resulted in a relatively conservative conclusion rather than an exaggerated one.

However, some limitations warrant consideration. First, although the majority of the studies adjusted for known risk factors for T2D incidence, such as age, BMI, smoking status, education, physical activity level and alcohol consumption, we could only retrieve age and BMI for all the studies and adjust only for them in our final model. The possible bias from residual confounders remained. Subgroup analyses that distinguish the studies with and without adjustment for these confounders would be informative. For example, we conducted the subgroup analysis for the studies adjusting for intake of cereal fiber; however, the overall RRs changed little, which were 0.892 (95% CI: 0.834, 0.954) and 0.916 (95% CI: 0.852, 0.985) for adjusted and unadjusted studies, respectively. Second, the magnesium intake in these studies were only assessed by FFQ, which do not capture the magnesium intake from drinking water and nutritional supplementation, and thereby might underestimate total magnesium intake and result in potential misclassification. However, the misclassification would most likely lead to an underestimated association. Third, influence of other nutrients or dietary components such as coffee [49], red meat [45], calcium [43] and fiber [14,19] that are correlated with dietary magnesium could not be excluded; other nutrients may have been responsible for the observed association partly or completely. Finally, publication bias may be a problem in our pooling analysis. Although we tried as much as possible to search for potential unpublished studies, no valid studies were identified from the available grey literature resources. However, the evidence (Harbord’s *p* = 0.401) did not indicate notable publication bias in our meta-analysis.

## 5. Conclusions

In conclusion, results from this meta-analysis indicate that dietary magnesium intake is associated with a reduced risk of type 2 diabetes (T2D). The greatest magnitude in risk reduction was found in the US population. A statistically significant linear dose-response relationship was identified across all the studies, and the largest magnitude association was found in the Asian population. A 100 mg/day increment in dietary magnesium intake was associated with an 8%–13% reduction in risk of T2D. No nonlinear dose-response relationship was found between incremental dietary magnesium intake and T2D incidence. Regarding the dose-response relationship between dietary magnesium intake and T2D in populations in Africa and Europe, more evidence is needed.

## Figures and Tables

**Table 1:** Characteristics of 25 cohort studies providing risk estimates for dietary magnesium intake and T2D incidence.

First Author, Year, Study, Country	No. of Case (Cohort Size)	Years of Follow-Up	Dietary Assessment Method	Case Ascertainment	Sex, Mean Age at Baseline (Years)	Magnesium Intake (Magnesium/Day) for Highest vs. the Lowest Quantile [RR (95% CI)]	Confounders Adjusted for
Hruby, 2014, FHSO, U.S. [7]	179 (2582)	7	Validated FFQ	Validated self-report	M and F, 53.9	395 vs. 236 (0.49 (0.27, 0.88))	Age, sex, energy intake, family history of diabetes, BMI, physical activity, smoking, alcohol, hypertension, dietary fiber
Oba, 2013, JPHCPS, Japan [50]	Men: 690 (27,769)	5	Validated FFQ	Validated self-report	M: 56.5	349 vs. 232 (0.85 (0.69, 1.06))	Crude *
	Women: 500 (36,864)				F: 56.9	356 vs. 211 (0.69 (0.54, 0.88))	
Hata, 2013, Hisayama, Japan [52]	417 (1999)	15.6	FFQ	Self-report	M and F, 57.0	215 vs. 133 (0.63 (0.44, 0.90))	Age, sex, family history of diabetes, BMI. HDL cholesterol, triglycerides, hypertension, smoking, alcohol, physical activity, total energy intake, carbohydrate, crude fiber, saturated fatty acid, polyunsaturated fatty acid and vitamin C
Weng, 2012, CVDFACTS, Taiwan [53]	141 (1604)	4.6	Validated FFQ	Self-report	M and F, 50.0	406 vs. 212 (0.38 (0.21, 0.70))	Age, sex, caloric intake, residential area, family history of diabetes, BMI, education, smoking, alcohol, physical activity, hypertension, hypercholesterolemia, hypertriglyceridemia, low HDL-cholesterol
Hopping, 2010, MEC, Hawaii [21]	Men: 4555 (36,255)	14	FFQ	Validated self-report	M: 57.4	185 vs. 129 (0.77 (0.70, 0.85))	Ethnicity, BMI, physical activity, education, calories
	Women: 4032 (39,255)				F: 57.2	200 vs. 139 (0.84 (0.76, 0.93))	
Kirii, 2010, JACC, Japan [23]	Men: 237 (6480)	5	Validated FFQ	Validated self-report	M: 53.3	323 vs. 156 (0.64 (0.44, 0.91))	Age, BMI, family history of diabetes, smoking, alcohol, physical activity, green tea, coffee, total energy intake
	Women: 222 (11,112)				F: 53.1	298 vs. 159 (0.68 (0.33, 0.75))	
Nanri, 2010, JPHCPS, Japan [24]	Men: 634 (25,872)	5	FFQ	Validated self-report	M: 56.5	348 vs. 213 (0.86 (0.63, 1.16))	Age, study area, BMI, smoking, alcohol, family history of diabetes, physical activity, hypertension, coffee, calcium intake, total energy intake
	Women: 480 (33,919)				F: 57.3	333 vs. 213 (0.92 (0.66, 1.28))	
Kim, 2010, CARDIA, U.S. [22]	330 (4497)	20	Validated FFQ	Validated self-report	M and F, 24.9	478 vs. 362 (0.53 (0.32, 0.86))	Age, sex, ethnicity, study center, education, smoking, alcohol, physical activity, family history of diabetes, BMI, blood pressure, total energy intake, saturated fat, crude fiber
Kirri, 2009, JPHCPS, Japan [43]	Men: 634 (25,877)	5	FFQ	Validated self-report	M: 56.5	331 vs. 245 (0.89 (0.72, 1.10))	Crude
	Women: 480 (33,919)				F: 57.3	314 vs. 248 (0.76 (0.59, 0.98))	
Villegas, 2009, SWHS, China [20]	2270 (64,190)	7	Validated FFQ	Self-report	F: 51.0	318 vs. 214 (0.86 (0.75, 0.97))	Age, energy intake, WHR, smoking, alcohol, physical activity, income, education level, occupation, hypertension
Schulze, 2007, EPIC, Germany [19]	844 (27,550)	7	Validated FFQ	Validated self-report	M and F, 49.6	359 vs. 298 (0.75 (0.60, 0.94))	Crude
Liu, 2006, WHS, U.S. [42]	651 (14,874)	10	Validated FFQ	Validated self-report	F: 54.5	340 vs. 307 (0.88 (0.76, 1.04))	Crude
Pereira, 2006, IWHS, U.S. [49]	1415 (28,812)	11	FFQ	Validated self-report	F: 61.3	334 vs. 281 (0.60 (0.47, 0.76))	Crude
van Dam, 2006, BWHS, U.S. [18]	1964 (41,186)	8	Validated FFQ	Validated self-report	F: 38.7	244 vs. 115 (0.65 (0.54, 0.78))	Age, energy intake, BMI, smoking, alcohol, physical activity, family diabetes history, education level, calcium, coffee, sugar, soft drink, processed meat, red meat
Pittas, 2006, NHS, U.S. [44]	4843 (83,779)	20	FFQ	Validated self-report	F: 46.1	313 vs. 281 (0.79 (0.64, 0.96))	Crude
Song, 2004, WHS, U.S. [45]	708 (14,924)	8.8	Validated FFQ	Validated self-report	F: 53.9	377 vs. 305 (0.47 (0.41, 0.55))	Crude
Song 2004, WHS, U.S. [17]	918 (38,025)	6	Validated FFQ	Validated self-report	F: 53.9	399 vs. 252 (0.88 (0.71, 1.1))	Age, smoking, BMI, exercise, alcohol, family history of diabetes and total calories
Lopez-Ridaura, 2004, NHS, U.S. [16]	4085 (85,060)	18	FFQ	Validated self-report	F: 46.1	373 vs. 222 ([0.73 (0.65, 0.82))	Age, energy, family history of diabetes, BMI, physical activity, smoking, alcohol, hypertension, hypercholesterolemia, other dietary variables
Lopez-Ridaura, 2004, HPFS, U.S. [16]	1333 (42,872)	12	FFQ	Validated self-report	M: 53.3	457 vs. 270 (0.72 (0.58, 0.89))	Age, energy, family history of diabetes, BMI, physical activity, smoking, alcohol, hypertension, hypercholesterolemia, other dietary variables
Hodge, 2004, MCCS, Italy [15]	365 (31,641)	4	FFQ	Validated self-report	M and F, 54.5	Per 500 magnesium increment (0.73 (0.51, 1.04))	Age, sex, country of birth, physical activity, family history of diabetes, alcohol, education, weight change, energy intake, BMI and WHR
Schulze, 2003, NHS II, U.S. [46]	741 (92,146)	8	Validated FFQ	Validated self-report	F: 36.0	341 vs. 281 [0.26 (0.20, 0.36))	Crude
Liu, 2000, NHS, U.S. [47]	1879 (75,521)	10	Validated FFQ	Validated self-report	F: 56.5	342 vs. 248 (1.04 (0.90, 1.19))	Crude
Meyer, 2000, IWHS, U.S. [14]	1141 (35,988)	6	FFQ	Validated self-report	F: 61.5	362 vs. 220 (0.67 (0.55, 0.82))	Age, total energy intake, BMI, WTH ratio, education, smoking, alcohol intake, physical activity
Kao, 1999, ARIC, U.S. [13]	White people: 739 (9506)	6	FFQ	Validated self-report	M and F, 54.2	418 vs. 308 (1.25 (0.88, 0.1.78))	Age, sex education, family history of diabetes, BMI, WHR, physical activity, alcohol, diuretic use, dietary calcium, potassium, fasting insulin and glucose
	Black people: 367 (2622)				M and F, 53.0	374 vs. 264 (1.05 (0.58, 1.93))	
Salmeron, 1997, HPFS, U.S. [48]	523 (42,759)	6	FFQ	Validated self-report	M: 53.3	461 vs. 262 (0.72 (0.54, 0.96))	Age, BMI, alcohol, smoking, physical activity, family history of diabetes
Salmeron, 1997, NHS, U.S. [54]	915 (65,173)	6	FFQ	Validated self-report	F: 46.1	338 vs. 222 (0.62 (0.50, 0.78))	Age, BMI, alcohol, smoking, physical activity, family history of diabetes

* Simple risk ratio without any adjustment.

**Table 2:** Pooled RRs * for T2D incidence of increased dietary magnesium intake by sex, area and adjustment.

Subgroup	No. of Studies (Dose Quantiles)	RR (95% CI)	*I*^2^ (%)	Heterogeneity-*p*
Sex				
Women	17 (58)	0.814 (0.774, 0.856)	82.4	<0.001
Men	7 (25)	0.838 (0.803, 0.876)	25.7	0.120
Both	7 (26)	0.854 (0.775, 0.941)	46.7	0.005
Area				
U.S.	16 (67)	0.817 (0.780, 0.857)	81.7	<0.001
Europe	2 (5)	0.858 (0.774, 0.951)	0	0.498
Asia	7 (37)	0.846 (0.811, 0.883)	10.2	0.294
Adjustment				
Adjusted ^†^	16 (76)	0.830 (0.806, 0.855)	39.6	<0.001
Crude ^‡^	9 (33)	0.808 (0.741, 0.881)	87.8	<0.001
Black people	2 (7)	0.815 (0.711, 0.935)	59.3	0.022

* Random-effects model was used; † Adjusted for age, BMI, energy intake, smoking, alcohol, physical activity, calcium, sugar, soft drink, red meat family and/or other dietary intakes, and/or family history, sociodemographic factors; ‡ Simple risk ratio without any adjustment.

**Table 3:** Estimated RRs for T2D incidence per 100 mg/day increment in dietary magnesium intake, adjusted for age and BMI.

	No. of Studies (Doses)	*I*^2^ (%)	RR (95% CI)	*p*-Value
All studies	25 (105)	69.72	0.916 (0.852, 0.985)	0.018
All studies *	24 (104)	69.13	0.882 (0.803, 0.969)	0.010
Sex				
Women	17 (56)	78.87	0.879 (0.756, 1.023)	0.094
Men	7 (23)	0	0.865 (0.767, 0.975)	0.020
Both	7 (26)	26.51	0.935 (0.853, 1.026)	0.148
Both *	6 (25)	29.00	0.857 (0.695, 1.057)	0.141
Area				
U.S.	16 (63)	79.09	0.910 (0.796, 1.042)	0.169
Europe	2 (5)	0	1.071 (0.264, 4.351)	0.644
Europe *	1 (4)	-	-	-
Asia	7 (37)	0	0.867 (0.768, 0.978)	0.022
Adjustment				
Adjusted	16 (72)	25.73	0.911 (0.864, 0.961)	0.001
Adjusted *	15 (71)	24.09	0.885 (0.830, 0.944)	<0.001
Crude	9 (33)	85.14	0.653 (0.462, 0.924)	0.018
Black people	2 (7)	0	0.747 (0.232, 2.409)	0.486

* One extreme dose was excluded.

## Abbreviations

CI	Confidence interval
FFQ	Food frequency questionnaires
HR	Hazard ratio
OR	Odds ratio
RCT	Randomized controlled trial
REML	Restricted maximum likelihood
RR	Relative risk
T2D	Type 2 diabetes
US	United States
